# Family members of deceased palliative care patients receiving bereavement anniversary cards: a survey on the recipient’s reactions and opinions

**DOI:** 10.1186/s12904-017-0199-7

**Published:** 2017-04-19

**Authors:** Swantje Goebel, Sandra Stephanie Mai, Christina Gerlach, Ulrike Windschmitt, Karl-Heinz Feldmann, Martin Weber

**Affiliations:** 1 Interdisciplinary Palliative Care Unit, III. Department of Medicine, University Medical Center of the Johannes Gutenberg University of Mainz, Langenbeckstr. 1, 55131 Mainz, Germany; 2 University Medical Center of the Johannes Gutenberg University of Mainz, Langenbeckstr. 1, 55131 Mainz, Germany

**Keywords:** Bereavement anniversary cards, Bereavement support, Palliative care, Bereaved family members, Survey, Questionnaire

## Abstract

**Background:**

Bereavement support is part of palliative care. Sending out bereavement anniversary cards is one intervention of follow-up support for the bereaved. This study evaluated the suitability of bereavement anniversary cards as an appropriate method in bereavement care.

**Methods:**

A questionnaire was sent to each card recipient since the starting point of this practice (October 2014-June 2015). Data was analyzed descriptively.

**Results:**

24 of 68 deliverable questionnaires were returned (response rate 35%). 22 out of 24 recipients felt pleased receiving the card. No participant felt annoyed on receiving the bereavement anniversary card; every participant agreed to at least one positive reaction (i.e. pleased, grateful or consoled).

**Conclusions:**

The participants’ reactions and opinions about receiving the anniversary card were decidedly positive and indicate the continuation of this practice. Those few less pleased reactions may be related to timing and the first anniversary of the patients’ death and therefore an expression of grief rather than a dissatisfaction with bereavement anniversary cards, as such.

## Background

In accordance with the World Health Organization, [1] palliative care does not end with the patients’ death, but includes supporting the family members in dealing with the loss. Bereavement support can help bereaved individuals adapt to their loss [2] and has become an established part of palliative care. Accordingly, various interventions of follow-up support for the bereaved have been implemented in the Interdisciplinary Palliative Care Unit at the University Medical Centre, Mainz, Germany, e.g. bereavement counselling, half-year memorial services, and grief brochures. In addition, bereavement anniversary cards are sent out to the closest relatives one year after a patient’s death. Objectives of the card include honoring the loss, expressing sympathy, and giving support at this presumed critical time.

While no recipient gave feedback on this service since it has been applied in October 2014, clarification was needed: What do bereaved family members feel and think about receiving our card? In principle, is sending out a card appropriate on the occasion of the first anniversary of the patient’s death?

Numerous studies have been conducted such as nationwide surveys [3–5] and facility specific case studies [6, 7], which examined comparable programs for bereavement support. Sending out bereavement anniversary cards is described as one common support practice. For example Mather et al. [3] found that 95% of Australian palliative care services have implemented various supportive strategies for the bereaved; for 53% of them sending out anniversary cards is common practice. Similarly in Europe, Keegan et al. [4] showed that 302 out of 370 facilities from 25 countries provided bereavement services, with 22% of them regularly sending out bereavement anniversary cards. However, Aoun et al. [8] highlight the lack of a detailed assessment of the benefits and risks of such services. A literature review revealed only one British study published in 1995 which specifically examined reactions to the card [9]; in this study 92% of the 85 recipients were glad to have received the card [9]. Still, several aspects of the bereavement anniversary card, such as the appropriate date of mailing, have not been examined.

In view of the rarity of studies in this regard, and the complete lack of comparable studies in Germany, we designed a survey in order to fill this gap and to assess the suitability of this support practice for our palliative care ward. This study inquired about the recipient’s reactions and opinions on receiving a bereavement anniversary card, in order to evaluate if this is a suitable support practice of bereavement care for the family members of deceased palliative care patients. The study was approved by the local institutional ethics committee.

## Methods

### Sending out of bereavement cards: Procedure and choice of recipients (study population)

The bereavement card is sent out by our department exactly one year after the patient’s death. Designed as a folded card with a nature motif, it consists of a handwritten salutation followed by a printed text denoting the recipient’s specific relationship to the deceased, and expressing our condolences and sympathy. Usually the person, who is listed as first contact person in the patient’s medical chart, is chosen as recipient of the bereavement card assuming that he or she is probably the closest relative or friend of the deceased patient. If there has not been any individually arranged follow-up support, the bereavement anniversary card is usually the second contact after the patient’s death (the first being the invitation to one of the biannual memorial services).

### Questionnaire-based data collection

A questionnaire consisting of four structured and two open-ended questions relating to this objective as well as the collection of demographic data was designed by a working group of those with medical, nursing, psychological, and chaplaincy expertise. Based on the questionnaire used by Hutchison [9], reactions on receiving the bereavement anniversary card were to be answered with a 4-point Likert scale (“Strongly agree”, “Agree”, “Disagree”, “Strongly disagree”, and the additional category “Can’t remember”). The provided statements represent a spectrum of reactions possibly provoked by the card. “I was disappointed” for example refers to the possibility that one could have expected a more personal approach. Further questions concerned the appropriateness of layout, text, and date of mailing one year after their relatives’ death. Two open-ended questions asked the recipients what they liked best and which adjustments they would suggest. In addition to every structured question a free text field offered the possibility for explanatory statements.

The questionnaire was mailed to each recipient of a bereavement anniversary card over the period from October 2014 until June 2015. A covering letter guaranteed voluntary participation as well as anonymization of data.

### Subsequent telephone interviews

Attached to the questionnaire a separate card invited the participants to take part in a subsequent telephone interview. The interview guide, developed by the working group, focused on further aspects of the bereavement anniversary card, examined the participants’ opinion on other activities of follow-up support, and investigated their needs and wishes regarding bereavement counselling.

Notes were taken as the interviews were conducted and from memory once concluded.

### Data analysis

The data were organized in Excel-sheets. The responses to the structured questions were analyzed descriptively. The answers to the open ended questions as well as the memory protocols from the telephone interviews were used to complement the descriptive findings, after having been analyzed in discussion within the research group. Due to the paucity of free text-answers and the decision not to audiotape the telephone interviews, a formal qualitative content analysis was not performed.

## Results

From 87 questionnaires, 19 were returned undeliverable. From the remaining 68 questionnaires 24 were returned completed (35% response rate). The sample included 14 spouses, seven adult children, one sister, one grandchild, and one friend of deceased palliative care patients. 10 recipients were aged inbetween 45 – 54 years, 4 inbetween 55–64 years, 7 inbetween 65–74 years, and 3 over 75 years. Subsequent to the postal survey, telephone interviews with six participants could be conducted. Duration of the interviews was from 25 to 55 min.

As presented in Table 1, 22 out of 24 recipients felt pleased receiving the bereavement anniversary card. For 22 recipients the card initiated a feeling of gratefulness. 21 responded that they felt consoled. None of the 24 participants felt annoyed upon receiving the card, likewise 23 have not been disappointed, and everyone strongly disagreed with the statement “It had no particular meaning to me.” Although every recipient agreed to at least one positive reaction (i.e. pleased, grateful or consoled) on receiving the anniversary card, nine recipients additionally reported on sad feelings. Furthermore two of those nine denied feeling pleased to receive the card and one of these two participants did not feel consoled.

**Table 1:** Respondent’s reactions on receiving the bereavement anniversary card

Response Options Items	Strongly Agree	Agree	Disagree	Strongly Disagree	Can’t Remember
I was pleased.	18	4	1	1	0
It made me sad.	2	7	5	9	1
I was annoyed about.	0	0	1	23	0
I was grateful.^a^	15	7	0	0	1
I was disappointed.	0	0	0	23	1
I felt consoled.	10	11	1	1	1
It had no particular meaning to me.	0	0	0	24	0

*N* = 24 (^a^ = 1 missing)

One participant, who did not feel pleased receiving the card, stated within the subsequent telephone interview that being pleased for him was not an adequate expression in conjunction with a day of great sadness. However, he valued the card as a gesture of compassion, especially since he received it on that specific day.

Table 2 presents the recipients’ opinion on the format of the card. 23 out of 24 participants estimated layout as well as text of the bereavement anniversary card as adequate.

**Table 2:** Opinion on format of the bereavement anniversary card

Response Options Items	Strongly Agree	Agree	Disagree	Strongly Disagree	Can’t Remember
Layout is adequate.^a^	18	5	0	0	0
Text is adequate.^a^	19	4	0	0	0
Card appears as clinical.^b^	1	1	1	16	2

*N* = 24 (^a^ = 1 missing; ^b^ = 3 missings)

All of the 24 participants estimated the date of mailing the card one year after a patients’ death as most suitable. Three participants specified that a) this is the first anniversary, b) at the first anniversary words of comfort feel good, and c) the card brings back memories of shared experiences. One participant suggested an improvement in the design of the card. Ten participants specified what they liked best, whereas four of them emphasized the caring and attentive purpose behind sending out bereavement anniversary cards. For example:

“It was a heartfelt gesture, which gave me comfort and built me up a little.”

”It’s this honoring and caring act of yours, even afterwards.”

”I like the way you keep the family members in mind.”

Regarding the format and text of the anniversary card within the telephone interviews, two participants endorsed its personal touch and underlined, how important it is to denote the recipients’ relationship to the patient in the text of the card and to write the salutation by hand.

Further possible activities of bereavement support were evaluated within the telephone interviews. Half-year memorial services were highly appreciated. All participants approved the ecumenical approach, and they emphasized the occasion to get in touch with the health care professionals again. All telephone participants stated that they hadn’t been aware of the information material concerning grief support which was available at the information board on the ward. Those two participants who sought consultation after the patients’ death arranged it independently.

## Discussion

None of the participants were annoyed or disappointed by the bereavement anniversary card. 22 out of 24 were pleased and 22 out of 23 were even grateful for receiving the anniversary card. Overall the participants’ reactions and opinions about receiving the card were clearly positive and indicate the continuation of this practice.

Every participant indicated at least one positive reaction on receiving the card, hence there is no indication against this practice. The current format, including the date of mailing exactly one year after the patient’s death, was also evaluated as appropriate. The findings underline the importance of personally designing the bereavement anniversary card, instead of sending out standardized cards.

Some participants showed simultaneously positive and less pleased reactions to the card. Hutchison examined similar findings within his study, as for 7% of the 85 participants the anniversary card was upsetting and 6% would rather not have received one, finding it upsetting or of no comfort [9]. The author summarized the reasons given for upset as appropriate expressions of grieving.

Less pleased reactions like being sad or not consoled may be increased by the grave occasion, as one participant, who did not feel pleased receiving the card, stated within the subsequent telephone interview. This statement corresponds with Hutchison’s results, whereby 86% of the 85 participants found the first anniversary particularly difficult [9]. Participants confirmation of the statement “It made me sad” might also be interpreted in this sense, since seven of the nine participants who agreed to this statement, simultaneously stated they felt consoled receiving the card. So being reminded can be simultaneously painful and consoling. However, it might be that those sad feelings are not primarily initiated by the bereavement anniversary card, but rather an inherent and reasonable part of the grieving process [10], and preoccupation with sad memories of the deceased being one of several expressions of normal grief [11].

These findings are limited by several factors. The study has been conducted at only one palliative care ward. Generalization is limited furthermore in terms of small overall sample size and a response rate of 35%, although comparable to response rates in other studies. Special consideration should be taken on the non-responders’ reasons for not participating in the survey, which remain unknown. It cannot be excluded that non-responders did not appreciate the bereavement anniversary card. Another shortcoming of this study is that the questionnaire has not been validated previously.

## Conclusions

All in all, our findings suggest that sending out bereavement anniversary cards is a useful support practice, intended as a caring and attentive gesture in addition to others. Further research on bereavement support interventions from health care professionals with larger samples is required in order to evaluate the effect on bereaved families. The data collection with semi-structured interviews provided a more thorough insight into individual perceptions and might be a basis for an extended follow-up survey covering a larger sample size.
